# Machine Vision for As-Built Modeling of Complex Draped Composite Structures

**DOI:** 10.3390/ma14030682

**Published:** 2021-02-02

**Authors:** Oliver Döbrich, Ayoh Anderegg, Nicolas Gort, Christian Brauner

**Affiliations:** Institute of Polymer Engineering, FHNW University of Applied Sciences and Arts Northwestern Switzerland, Klosterzelgstrasse 2, 5210 Windisch, Switzerland; ayoh.anderegg@fhnw.ch (A.A.); nicolas.gort@fhnw.ch (N.G.); christian.brauner@fhnw.ch (C.B.)

**Keywords:** composites, digital-twin, as-built, finite elements, machine vision, fibers, textiles, draping

## Abstract

The transition in the use of fiber composite structures from special applications to application in the mass market is accompanied by high demands in quality assurance. The consequential costs of unclear process design, unknown fiber orientations, and uncertainty regarding the effects of any fiber angle deviations can lead to market considerations (higher costs/time for development) in mass production that advise against the use of fiber composites, despite their superiority compared with conservative materials. Active monitoring of the deposited reinforcement layers and an evaluation of the real fiber orientation can form the basis of a robust industrial use of fiber composites by a first-time right production that is able to reduce the process variability. This paper describes the application of an image analysis system to provide both geometric topology and local reinforcement fiber orientation feedback to a finite-element (FE) model. The application during an industrial composite part production is described, and the possibilities of using it for the improvement of the lightweight character, the reduction of rejects, and the realization of a quality management system are shown. The determined component data are made directly available for use in numerical simulations and, thus, they serve as a non-destructive evaluation of the components under real conditions in which all production-dependent influences that affect the fiber orientation are incorporated.

## 1. Introduction

**Figure 1:**
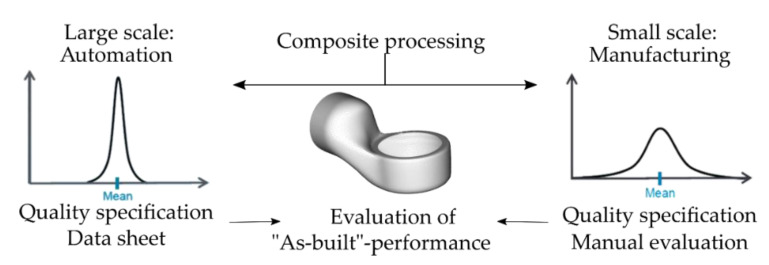
Quality distribution of composite parts in the production method.

As the production of complex-shaped composite structures is a sequential process characterized by either manual or automated handling of unstable reinforcement fabrics, deviations from an ideal “as-planned” configuration can be observed. These deviations may vary in terms of the chosen process and material, as illustrated in Figure 1, but they are never to be neglected. The design of composite parts is regularly carried out with only a small reserve in the mechanical capacity. Deviations in the fiber orientation and defects from the manufacturing process may result in reduced structural stiffness and strength or unpredicted failure. For this reason, conservative design concepts are mostly applied that are in conflict to an optimal lightweight design. Therefore, the use of machine vision to digitalize the quality assessment of the composite production is suggested.

The digitalization of composite materials, textile constructions, and inhomogeneous structures in general is driven by the development of simulation models and the trends in digital design and manufacturing. The local material behavior of composite materials can be obtained from virtual predictions (forming simulations [1,2,3], drape simulations [4,5,6], flow simulations [7,8,9], or process-induced stress prediction [10,11]) or from destructive (microtome sections [12,13]) or non-destructive material examinations (computer tomography [14,15,16], eddy current analysis [17,18], thermography [19,20], or X-ray analysis [21,22]) on existing parts.

**Figure 2:**
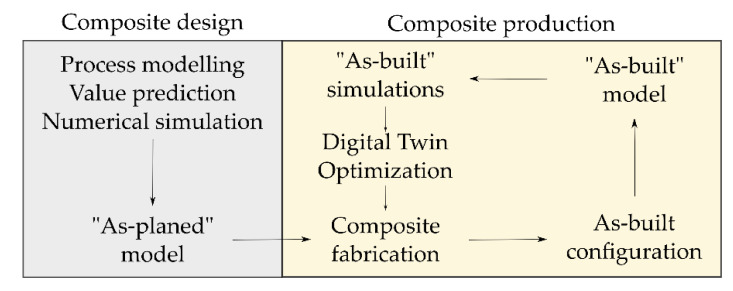
Schematic explanation of the as-planned and as-built configurations for composite modeling.

Digital methods are used for the designing of structures; therefore, an ideal as-planned configuration is obtained. This configuration can be varied [23] or even further optimized by virtual methods [24,25]. Following the variations found in both the manual production process and the industrial process guidance (Figure 1), the actual resulting structural part exists as a variation of the ideal as-planned configuration within the limits of the process-dependent deviation in a representative “as-built” configuration. The examination of this configuration and its transition into a Digital Twin for the usage of adapted simulation models is desired. Research has been carried out to adapt to these process deviations by variations of the virtual models, as described in [23]. The possibility to conduct simulations with a specific set of local material properties can improve the composite development and the quality of the virtually obtained results for composite behavior. This is illustrated in Figure 2.

Several models on fiber-reinforced composite materials have been used on different length scales to describe the geometrical construction and to predict mechanical behavior. Those are not covered within this publication; however, models that predict the orientation and fiber distribution in a final structural preform or part affected by the source material and process influences will be mentioned. These virtual models were developed to predict the inhomogeneous fiber orientation of fiber-reinforced composites for short fiber-reinforced composites regarding injection molding [26,27] and compression molding [28,29] as well as continuous fiber reinforcements on the micro [30,31], meso [32,33,34], and macro level [9,35,36,37] for both dry fabrics and pre-consolidated organo-sheets to obtain as-planned configurations suitable for the calculation of the structural stiffness or strength.

The results obtained by these models are suitable for use with an as-planned modeling approach. The results of the introduced models aim for prediction of the local fiber orientation in several composite-forming processes. These models include the process parameters and material specific input data. They represent a single possible and idealized as-planned configuration of the structure in question.

Regarding the design of a model following the as-built approach, efforts have already been made to provide fiber detection systems for the analysis of the real fiber orientation existing in composites aiming for a Digital-Twin configuration of a single available structure. In this sense, the definition of a Digital-Twin is a representation of each manufactured part in a virtual environment. To evaluate the local fiber orientations, microtome slices have been made from composite materials to examine the structural construction; however, the method is of a destructive nature [38].

Micro CT scans have been used to build composite unit cell models to predict mechanical properties [16]; however, the application on a global structural level is unknown. Eddy current sensor data can be used to evaluate the results of the preforming process [18]. This method is applicable to large areas of composite structures and is able to deliver the local fiber orientation in the real state but is only applicable to carbon fibers or other conductive reinforcement materials since conductivity is crucial to this method.

Camera images have also been used for mapping the fiber orientation in numerical composite models [39]. Thermography methods have also been used to determine the local fiber orientation in composite materials [19,40]. As with the other introduced methods, such as eddy current examination, the thermographic method aims toward an investigation of the finished composite product. Information on the local fiber orientation and the layer to which it is assigned must be evaluated with additional expense.

The possibilities to use the data are generally restricted by the effort of capturing and the availability at the site. Results from small samples correspond to the local structural geometry only and are not applicable on a structural level. Destructive examinations are also not desirable. However, their data can be used for the validation of computational models in discrete states. The prediction of anisotropic material behavior from computational models is part of the ongoing research [31,41,42] as well as the mapping of actual material properties into models used in virtual simulation [43].

The availability of the captured data, regardless of the capturing method, must be ensured in a virtual environment. The usability of real-production data within digital databases was covered in [44]. In this contribution, a technological approach is introduced that relies on image processing methods to identify the local fiber orientations during the preforming process. The local information regarding the fiber orientation is monitored and evaluated during the production process through the optical examination of each layer. Therefore, no restrictions on the material and its physical or mechanical properties are made, if the surface texture is visible. Additionally, the process does not influence the production at all. The used carbon-fiber prepreg ensures the preservation of the captured state during the consolidation process due to its rigid and sticky nature. The data captured by machine vision is processed and evaluated to obtain information regarding the actual condition of composite parts after their fabrication. An approach is presented regarding importing the data back to the initial numerical design model by a standardized file format to replace the assumed fiber orientations, which are used in the parts design process, with real production data from every single manufactured part. This enables all the beneficial methods known from Digital Twin applications, such as optimization, quality assurance, and life performance analysis.

## 2. Materials and Methods

### 2.1. Composite Reinforcement Fabric

The carbon fiber prepreg Krempel KGBX 2508 HT 3K by the Krempel Group (Vaihingen, Germany) was used for the presented studies. Composites from this Twill woven fabric are characterized by mechanical properties along the main reinforcement directions 1 and 2 (determined according to DIN EN ISO 527-4) and in the diagonal direction 12 (according to ASTM 3518) found in Table 1.

### 2.2. Apodius Vision System 3D by Hexagon

**Figure 3:**
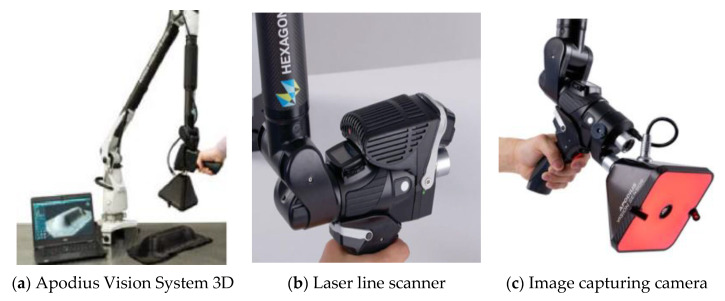
Apodius Vision System 3D by Hexagon (**a**) with laser scanner (**b**) and camera (**c**) [source: Hexagon].

The Apodius Vision system 3D by Hexagon (Figure 3) was applied for the presented examinations. The system comprises HP-C-V3D Vision Sensor hardware—designed to fit to an Absolute Arm with RS5 Laser Scanner—together with the custom-built Explorer 3D software package. This was used for capturing both the structural topology of the part as well as the surface images that are further processed to obtain information on the local fiber orientation. The topological information provided by the system can be used for several investigations, as shown in Section 2.3.

However, the introduced method of building a digital twin with the Hexagon system does not generally require these measurements and can be carried out without the scanning of the part by the laser line scanner. The presented method may be carried out with the surface images mapped on geometrical information provided by a computer-aided design model (CAD) instead as well. Additional opportunities in using real captured topology data instead of idealized CAD data are shown in the following subsection.

### 2.3. Generic Robotic Limb Joint

**Figure 4:**
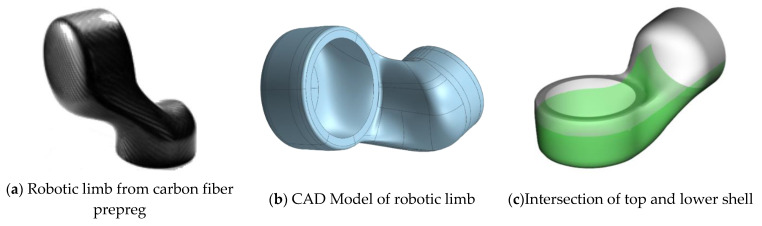
Robotic limb joint.

The structural geometry used for the investigations presented was provided by the company Nägeli Swiss AG (Güttingen, Switzerland). The generic limb joint (Figure 4a) for use in robotic applications is composed from two intersecting shell structures that are joined by a stepped lap (Figure 4c). The dimensions of the limb were 180 × 180 × 150 mm^3^ (H × W × T). Along with the relatively small size and the harsh edges with small radii, the capturing and evaluation of the shown part is challenging due to reflections and strong changes in the local fiber directions.

**Figure 5:**
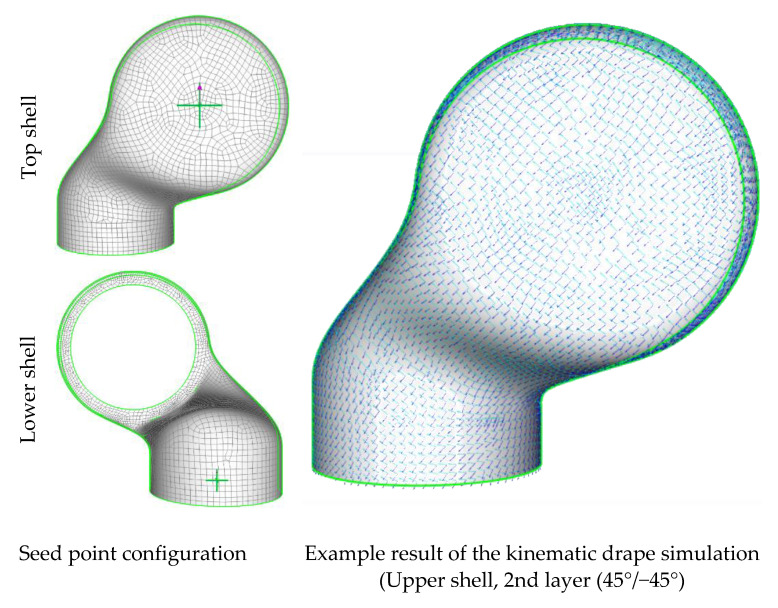
Procedure and result of the kinematic drape simulation for the as-planned configuration.

The robotic limbs used in the presented studies were manufactured by handy layup and manual draping of the two shells as illustrated above. The main orientations of the prepregs when draped onto the limbs are given in Table 2. The values refer to the orientation in the seed point according to Figure 5. The limb has been cured in accordance to the prepreg supplier guidelines to reach a complete cure of the resin. The achieved degree of cure as well as the final fiber volume content are given in Table 2.

### 2.4. As-Planned Data

For the provision of the as-planned dataset, which can be used for the initial composite design of the structural part, ANSYS Composite PrePost (ACP) was used to create the fiber orientation from a kinematic drape simulation. This process includes the structural geometry as well a seed point that corresponds to the starting point of the fabric deposition process. The simplifications and restrictions of the kinematical drape algorithms do not take all possible influences into account but are consequent for most reinforcement fabrics, such as the pre-impregnated weave used for the investigations in this study.

The draping seed point for the kinematic drape simulation was set to the point where the fabric was initially placed on the mold for the manual hand layup used to produce the robotic limbs. This seed point is visualized along with an exemplarily result of the kinematic drape simulation in Figure 5. The as-planned result is used as the target for the manual draping to achieve a minimal deviation for the as-built configuration.

### 2.5. As-Built Data

The main difference in comparing as-built data with an as-planned dataset is the uniqueness of the as-built set, since it references only the structure from which the data was retrieved. The as-planned data has a general character and represents a mean result, including the process parameters and the material input (depending on the way of generating the as-planned data), whereas the as-built dataset includes everything from deviations in the processing to deviations in the material. The real existing state is captured and processed. Therefore, statements and results obtained using the “as-built” data can only be accounted on the structure from which it was retrieved.

#### 2.5.1. Ply Wise Scanning of Preform Geometry

The Apodius Vision system can be equipped with a laser line scanner as shown in Section 2.2. This scanner enables the virtualization of structural geometries with a high level of details due to the excellent scanning resolution of <1 mm per point. The parts topology must be scanned by hand by hovering the scanner over the desired geometry. The laser line is always visible, and the scanning result can be seen in the Apodius Desktop environment at any time to check for missing spots and the achieved results.

As already mentioned, the processing of the surface mapping and the evaluation of the local fiber orientation (as shown in Section 2.5.3) can be performed using an ideal CAD geometry of the structural part. However, the ply wise scanning can improve the process of mapping for thick parts, and additional information regarding the local thickness of each layer can be obtained. The investigations presented in this paper have been done by mapping the surface images (Section 2.5.3) onto the CAD data of the part. The ply wise scanning was done to evaluate changes in the layer thickness. The thickness information can be calculated by determination of the distance from one ply to another, at certain points, such as the center of the discrete triangular element surface mesh. This procedure and the results are illustrated in Section 3.1.1.

#### 2.5.2. Scanning of Global Structural Geometry

The laser line scanner can be used not only to provide the geometrical model of every single layer while preparing the composites preform but also to scan the resulting structural part after preforming and after curing. The outer shell surface was scanned in this regard. The result can be used for quality assessment when compared to the initial CAD geometry. Unsymmetrical layups and complex geometries are known to provoke inhomogeneous thermal and chemical shrinkage during the consolidation.

With a virtual scan of the final part, examinations regarding these structural deformations can be carried out. For demonstration purposes, an unsymmetrical limb was manufactured as shown in Table 1. The obtained point cloud from scanning both the limb manufactured with symmetrical layup as well as the point cloud obtained by scanning the unsymmetrical layup were compared with the initial CAD geometry by determining the Hausdorff distance [45] of the initial CAD and the scanning result (Section 3.1.1).

#### 2.5.3. Evaluation of the Local Fiber Orientation

**Figure 6:**
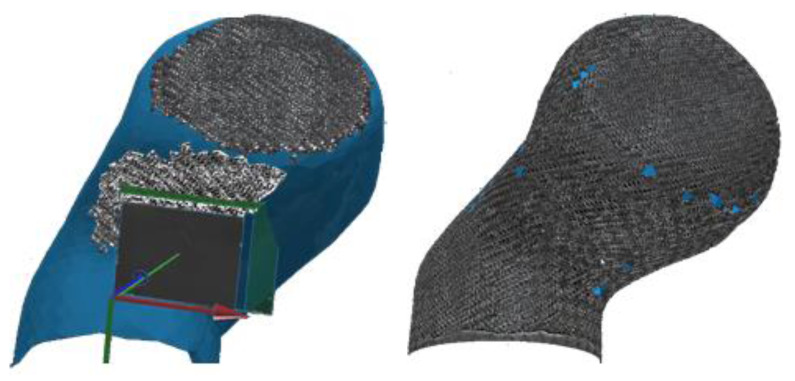
Capturing and mapping of surface images onto the structural surface mesh.

To evaluate the local fiber directions, images of the actual drape result were taken and mapped virtually onto the surface mesh, which was provided as a result from geometrical scanning or from the ideal CAD data. Here, the assumption is made that all fibers within a technical yarn are ideal parallel aligned. In this way, the evaluated fiber direction can be assigned to a local place on the structural part by evaluation of the yarn orientation. This procedure is shown in Figure 6.

**Figure 7:**
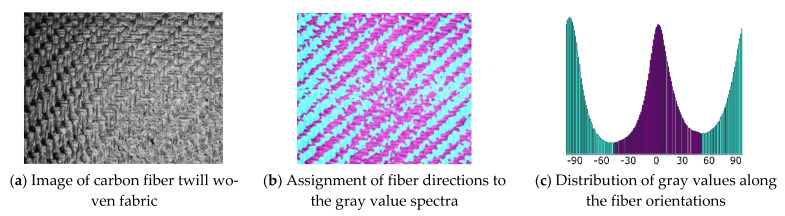
Material teaching and the assigning of fiber directions due to the gray value distribution.

To assign the fiber directions correctly by vision analysis, a reference image of the material must be taught to the software. This is done by providing a picture of the material as shown in Figure 7a. Since every region with alike properties has the same optical appearance, the gray values of the picture are analyzed, and every local region is examined by texture segmentation algorithms [46]. This edge-oriented method is beneficial for fabric textures as for most of the commonly used fiber reinforcements. Every segment is accounted with a corresponding reinforcement direction. This step must be done manually by the user. Afterwards, the software can account for the local fiber orientation according to the found gray value within the spectra (Figure 7c). Since the position of the camera system is known by the software, every captured image can be assigned to a position in space, and the region on the structural surface is known. With this information, the local fiber orientations can be mapped onto any mesh independent of its mesh size.

**Figure 8:**
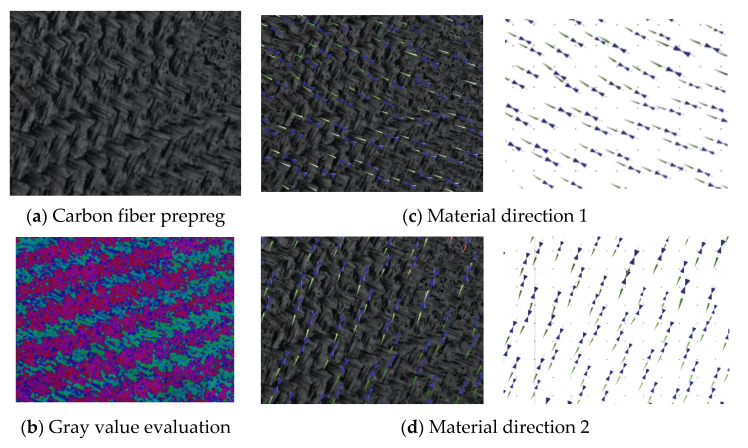
Evaluation of the gray value distribution and assignment of material directions.

The results of the image capturing procedure are displayed in the Apodius desktop environment in real time, and blind spots can be identified. As an example, Figure 8 shows the use of the system.

### 2.6. HDF5 File Format

For the transfer of the visually determined properties into commercial FEM programs such as ANSYS, the file format HDF5 was established. This is a hierarchically structured data model for storing and managing data. This makes it sufficient for storing the captured information regarding the discretized geometry (nodes and elements) and the local material properties (thickness and fiber orientation).

**Figure 9:**
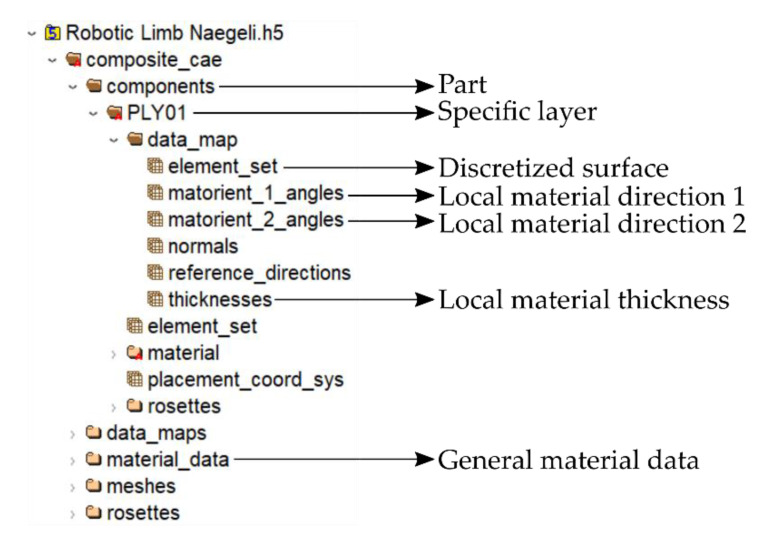
HDF5 file structure with discrete data on material and geometry.

The position of the discretized geometry in space, the orientation of the material directions and other parameters, such as the layer thickness, local density, and the general material properties, can be stored. Then, the data can be retrieved, extended, or manipulated as needed. This is not limited to data types or data sizes and, thus, it allows the efficient and flexible processing of complex data. An example of the HDF5 file structure that is suitable for import into ANSYS is shown in Figure 9.

ANSYS ACP makes it possible to import this information into an existing workload to be able to extend a created finite-element (FE) model with real recorded data of an existing component (as-built). The material fiber orientation as well as the local material thickness is interpolated and mapped onto the existing FE mesh, and the real-world data is utilized for virtual simulations.

### 2.7. Structural Bending Test

**Figure 10:**
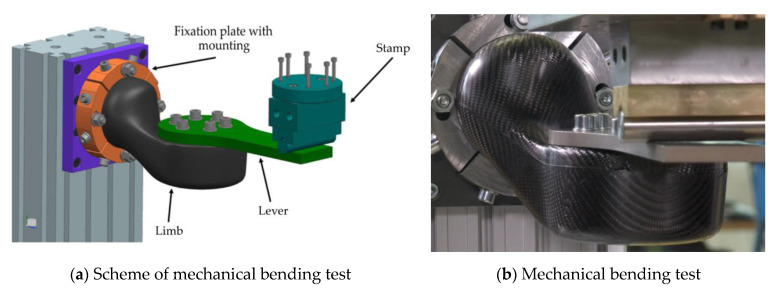
Illustration of the structural limb bending test.

An important aspect of this work is to prove the accuracy of the material properties created by the import of real fiber orientations. Therefore, a two-point bending test was chosen to test the fabricated limbs, and we compared the results with numerical simulations done with the as-built datasets. As shown in Figure 10, the whole test setup consisted of a fixation plate and a steel lever that was used to load the limb in a bending case scenario. During the bending test, a stamp was moved against the lever with a velocity of 5 mm/s to remain in a quasi-static range. The resulting displacement and reactional force were recorded by the used tensile test machine Zwick/Roell Z100.

## 3. Results

### 3.1. Modeling

#### 3.1.1. Scanning

**Figure 11:**
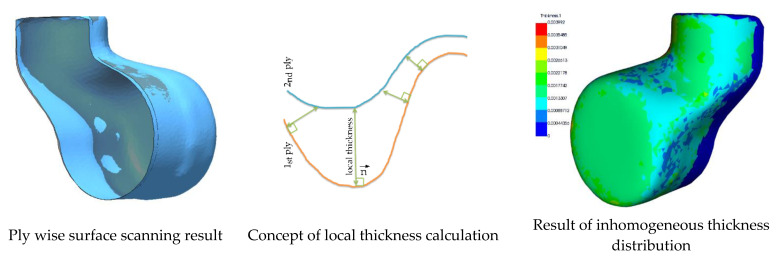
Determination of local ply thickness from laser line scanning result.

The distance of every element in a single layer to a certain (nearest) element in the next layer can be determined by calculating the surface to surface distance following the normal vector of the triangular element in question. As shown in Figure 9, this information is stored within the HDF5 file or can be retrieved from stl surface files. As an example, Figure 11 shows the determination of the local fabric thickness obtained from calculating the distance of the first reinforcement ply (45°/−45°) to the second ply (0°/90°) for the top shell of the limb. As for the data following the as-built concept, the calculated thickness only applies for the specific limb and the very specific layer.

The inhomogeneous thickness distribution illustrates the resulting local thickness as a function of the topology and material deformation since the radii and areas of higher shear strain influence the fabric thickness. However, the limb production process induces pressure on the stack of layers, and the thickness of each layer is reduced due to compressional forces and resin flow. Therefore, the information on the local thickness retrieved from the scanning of dry plies or pre-impregnated fabrics during the preforming cannot be used directly in a simulation of the consolidated part. The areas of increasing thickness and the relative fraction that each layer contributes to the resulting thickness can be determined.

**Figure 12:**
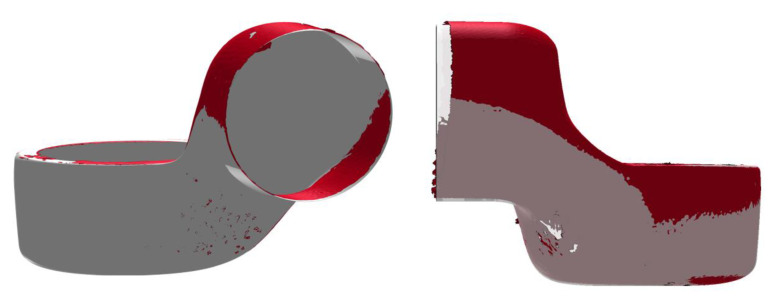

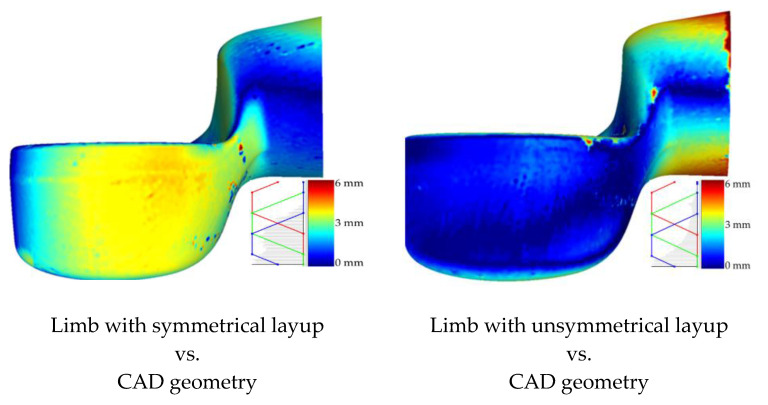
Comparison of two meshes by determination of the Hausdorff distance.

Regarding scanning for the final consolidated part, the results from the scanning of a symmetrical and an unsymmetrical part (Table 2) are shown in Figure 12 along with the depiction of the Hausdorff distance.

The unsymmetrical fabricated limb experienced greater deformation on the neck due to the chemical shrinkage of the matrix resin. The L-shape of the structure induced a movement from the larger bottom part toward the neck of the limb. The symmetrical structure remained in shape despite the complex geometry. The results from this analysis only have qualitative character, since the relative alignment of the measured model and the ideal CAD model was done manually by hand.

#### 3.1.2. Assignment of the As-Built Fiber Orientation

The information regarding the actual as-built fiber orientation must be captured for every part and every single ply, as explained earlier. These data must be imported ply-wise to the numerical model. For the investigations presented in this study, the composite simulation model *Ansys Composite PrePost* was used. This software package allows for a ply-wise description of composite materials according to an approach following the actual stacking similar to the composite structure manufacturing process.

**Figure 13:**
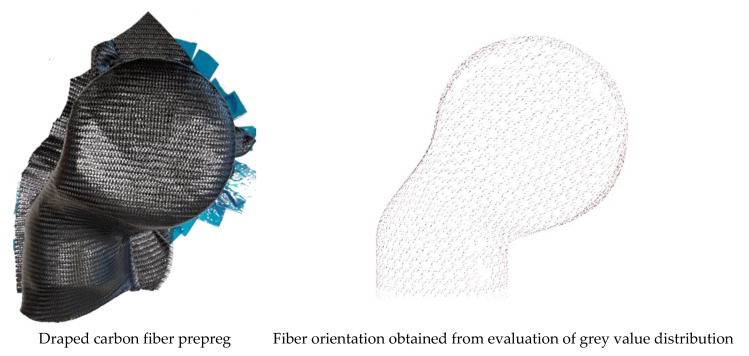
Results from the manual draping and evaluation of fiber orientation for the 0° ply (top shell).

Additionally, ACP supports the import of ply data using the introduced HDF5 file format. As the discretized mesh used for numerical modeling is not compulsorily identical to the mesh used for fiber orientation evaluation, the data must be mapped onto the FE mesh. To preserve the information obtained by the Apodius gray value evaluation, the target mesh size should be the same size or even smaller than the source mesh size. The results obtained by the Apodius gray value evaluation are shown for a representative layer in Figure 13.

**Figure 14:**
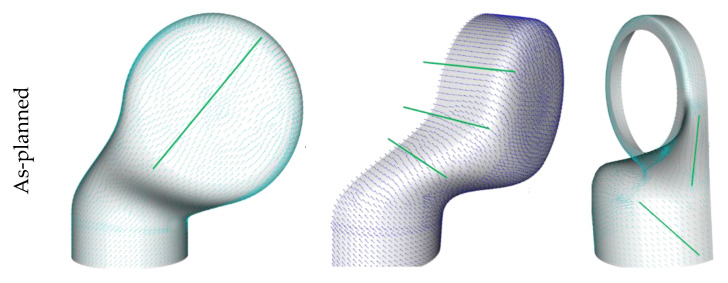

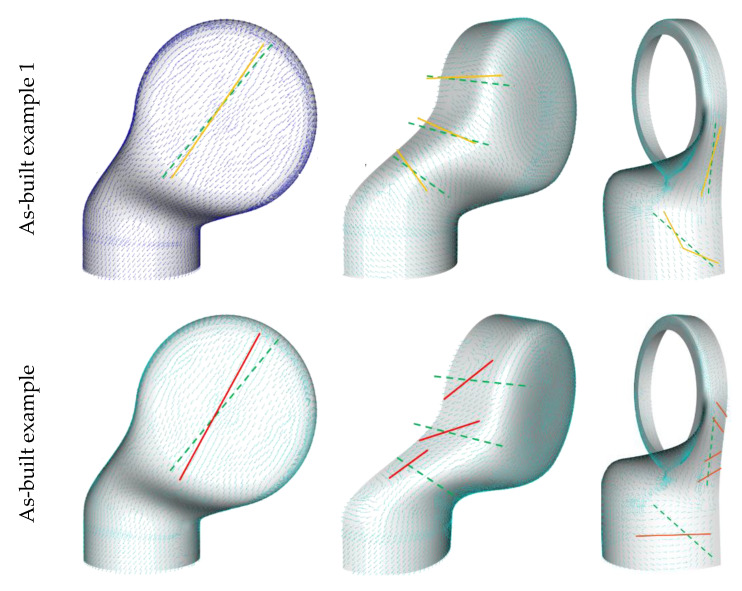
Comparison of the as-planned and two different as-built configurations.

The fiber orientations obtained from the manual drape process are compared to the target as-planned configuration at discrete points in Figure 14. The general orientations of the fibers are indicated by the visible lines. Since the limb geometry is very complex and pre-impregnated fabrics are hardly drapable, a significant deviation from the aspired result can be observed for some layers.

The results from Figure 14 are exemplary. The presentation of all the obtained results from each ply is not possible in the frame of this publication. However, a procreation of errors can be seen from the shown as-built configurations. The dataset found in example 2 had a larger deviation from the aspired 45° orientation. The complex geometry leads to a significant displacement of the fibers. Therefore, the obtained simulation model deviated significantly from the as-planned model. The simulation result used for the composite design process is not representative for the present part, and its condition and would not result in usable findings.

### 3.2. Two-Point Bending Test and Structural Simulation

#### 3.2.1. Mechanical Limb Bending Test

**Figure 15:**
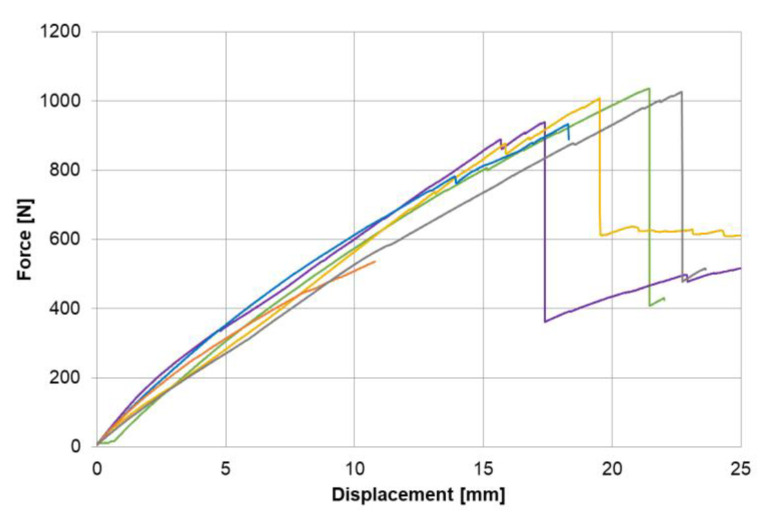
Results of the mechanical two-point limb bending test.

The produced limbs that delivered the as-built data were mechanically tested, and the resulting displacement–force curves were recorded. While most limbs could bear a maximum force of around 900 N, one limb already failed at 535 N. A deficit in the limb stacking was identified as the reason. The results are shown in the plot found in Figure 15.

**Figure 16:**
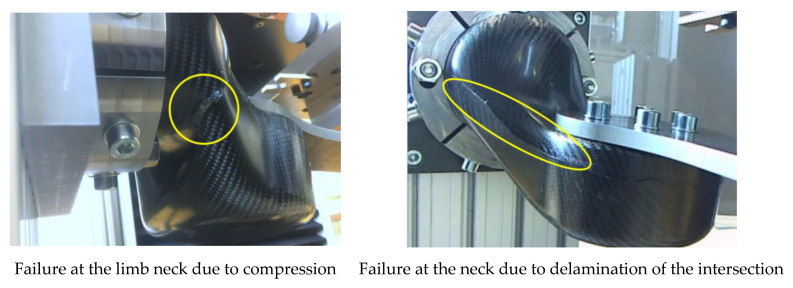
Observed failure modes from the two-point bending test.

As shown in the force–displacement plots, the final failure was preceded by preliminary damage. An evaluation of the failed part could provide information on the nature of the preliminary damage; however, a virtual evaluation was only possible for models that were processed following the as-built approach and included information on the actual structural properties. Additionally, different failure modes could be observed during the mechanical limb tests. They are shown in Figure 16. An investigation using a numerical model in the as-planned condition will always lead to the same result and would not be sufficient to analyze deviating parts.

#### 3.2.2. Numerical Simulation of Limb Bending Test

**Figure 17:**
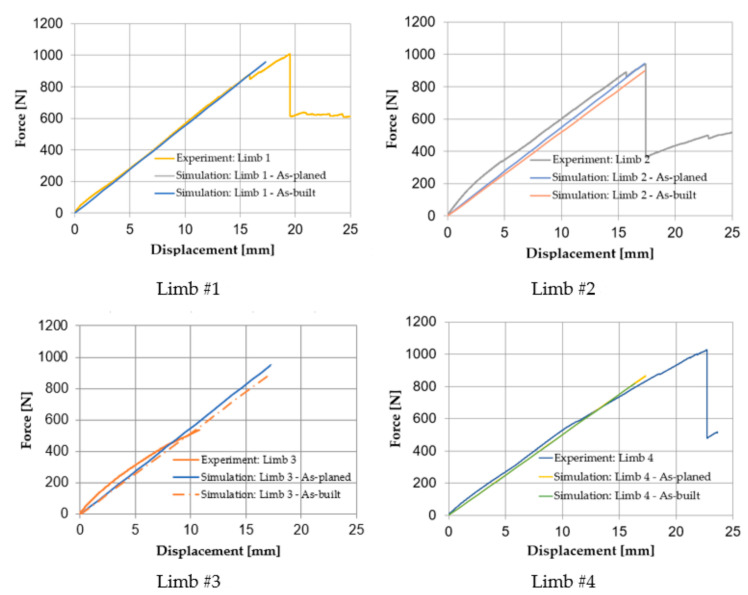
Direct validation of the as-built simulation models by comparison with the as-planned data.

A numerical simulation was conducted to analyze the stresses and strains during the bending deformation. Due to the number of as-built configurations, a variation of the force–displacement curves were obtained. The graphic plots in Figure 17 show that the as-planned configuration was stiffer than the as-built configurations. This is due to draping errors, local fiber deflections, and a general idealization of the as-planned configuration.

**Figure 18:**
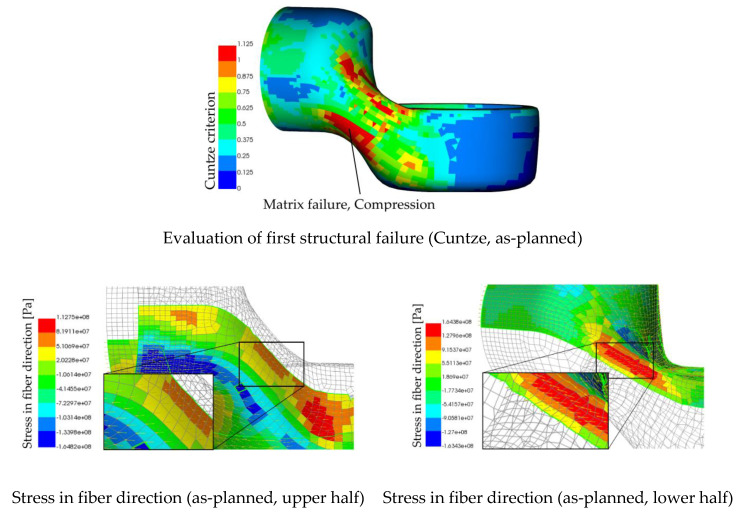
Stresses and result of load evaluation according to Cuntze for as-planned configuration.

The composite model was set up according to [47] where two individual unidirectional plies are used to model every single fabric-reinforced layer. The fiber orientations, derived from evaluating the image data of the surfaces, are mapped in a unidirectional manner on the UD plies with half the original plies thickness. For the failure investigation, the Cuntze and the Puck Criterium [48,49] was used to evaluate the local composite failure. A prior investigation of the as-planned configuration, which was loaded according to the presented two-point-bending case, is shown in Figure 18. The areas with the highest structural load, according to Cuntze, are visible. The fiber directions, as retrieved from the kinematic drape simulation, are visible in the plots that show the stress value in fiber direction for the corresponding positions.

The initial failure, as predicted by the as-planned simulation, can be found in two different areas located at the limps neck by nearly the same value for the Cuntze Criterion. Since compression is found on both sides of the neck, a geometrical nonlinearity as a result from a buckling deformation is assumed. Since the used material model does not include failure, an evaluation beyond this initial failure indication cannot be carried out. The stress plot (Figure 18) is indicating that compression in the fiber direction is the reason for a Cunze Criterion > 1 that displays a failure due to exceeding the matrix compressional strength. A prediction of the initial failure location is hardly possible, but both areas are to be concerned.

**Figure 19:**
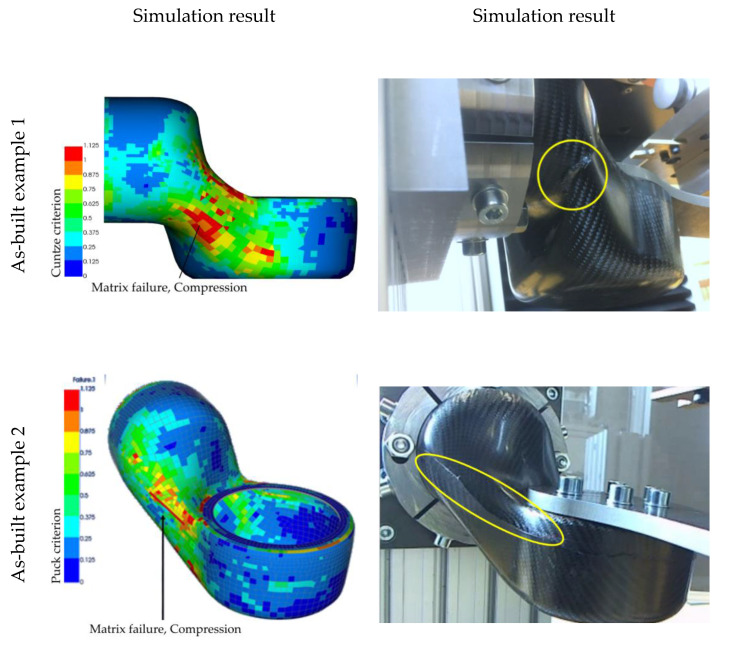
Results of the two-point bending simulation for as-built configurations.

As mentioned earlier, the manufacturing of the limbs led to different failure modes. We observed that the configurations that deviated only a little from the as-planned configuration failed in the same way as the as-planned simulation predicted (Figure 19). Configurations that deviated a lot, as seen in Figure 14, failed by delamination of the two jointed shells. Following the findings from the simulations, this may be due to compressional forces in the longitudinal direction of the segment joint area (Figure 4). Following the as-built approach, a validation of the as-planned setting was not possible, since all manufactured limbs deviated from this configuration. However, the results from the numerical bending simulation using the as-planned configurations can be found in Figure 19. As representatives for the as-built configurations, the datasets shown in Figure 14 were used, since these examples were used as variants for small and strong deviations.

By using the as-built datasets for the structural simulation, different locations of failure can be observed. The as-planned dataset is giving one result only, even though the difference in the structural construction, resulting mainly from manual handling and fabrication, could be shown (Figure 14). The analysis of the simulations using as-built datasets do match the later observed failure mode well enough to differentiate between them. The area that exceeds the materials strength is larger in the simulation results from example 1, whereas the as-planned results indicate two possible first failure locations. In addition, the result from example 2 could predict the exceeding load in a area where failure actually appeared. The fiber orientation of example 2 deviated a lot from the aspired as-planned configuration, as shown earlier. Therefore, a completely different load distribution was achieved, and different failure could be observed. The use of real product as-built data enables a prediction such as this. Comments on the classification of this result and recommendations regarding its use can be found in the next section.

## 4. Discussion

The need to develop a digital twin is evident in many aspects. The improvement of quality management, the reduction of rejects, and a further possibility to use virtual methods and models for the improvement of composites are only a few reasons. The increase of the lightweight character pushed further, and the competitiveness of the material also increased.

One possibility of transferring real material properties into a virtual environment is the optical capturing of fiber deposition states, their evaluation, and their transfer into known and established simulation tools. The Apodius system is based on captured images and has proven to be suitable in this regard. The topological scanning capabilities are helpful and can be used conditionally for analyses, such as local thickness evaluation. An analysis of defects or flaws is also conceivable. However, this only makes sense if the defects cannot be detected by the camera that is required. Otherwise, it is an additional step that adds time and costs.

Image acquisition in manual mode, as used in this project, is also complex and time-consuming. Nevertheless, the process is worthwhile, as the speed of composite production processes certainly allows for visual inspection with digital tools. An automated analysis can certainly capture the process without having to follow the speed of the measuring system during production. This was not the case in the manual measurements carried out. Here, the production time was equal to the data acquisition time, and the production had to be interrupted for the manual data acquisition.

The evaluation of the fiber angles within the process also involves the risk that the fiber orientation may still change during the process. However, an optical evaluation makes sense, as it is intuitive and can happen on a low-tech level. For safe process control, care and measures should be taken (the use of prepreg, binders, etc.). However, the application of such a vison system to a prepreg material is challenging compared to dry fiber fabrics. The resin residuals on the fabric surface decrease the contrast of the edges and/or increase the effects of the reflection of light. The illumination and the surrounding disturbance of the light can influence the quality of the measurements and should be as diffuse as possible.

The transfer of the recorded data into a standardized format was reliable. Within the project, it was possible to work closely with ANSYS Switzerland to enable the transfer of the data into an existing workspace, which is known as a valuable computational tool. The transfer of data within different software solutions is becoming increasingly important. Being able to use the specializations of individual software tools with a simple transfer of the models holds great potential for the use of virtual tools for digital composite development.

According to the idea of a digital twin, the tested limbs cannot be assigned to the as-planned configuration but only to the respective models. However, the totality of the mechanical test results with the confidence interval provides variations with which the as-planned configuration can be accounted for. Despite the as-built dataset being only relevant for the simulation of a very single structure, it enables the non-destructive simulation and prediction of stiffness, strength, or even durability without actually testing the present structure. Quality assessment methods are enabled, and a failure of the part during its lifespan can be traced back to its initial structural condition. The use of this technique in this regard requires well-fitted and validated simulation models.

The comparison of the results obtained with the as-planned and as-built models compared to the reality was good. The deviation was small for all observed cases and samples. During the examinations, an attempt was made to replicate the as-planned configuration. Therefore, large deviations were prevented. Despite the deviations in the layer structure, almost equal forces were achieved with the mechanical tests. However, different first ply failures were observed. The indicators for their occurrence could be predicted by the created as-built models.

A statistical comparison with the occurrence remains difficult, as the as-built configurations would have to be reproduced several times in an equal manner for this investigation. The presented results are not suitable for investigations regarding the used production process. However, in general, the presented study shows that parts manufactured with non-neglectable deviations can also bear the required loads as specified by the as-planned load level. This can be controlled and determined with simulation methods. This can reduce the amount of decisions for classifying products as committee parts. However, within this publication, emphasis was placed on the method demonstration and not on the interpretation of the simulation results. Therefore, the used model does not account for failure or stress redistribution effects after local cracks. The method is independent of the used simulation model.

## 5. Conclusions

By using the image analysis system and the ANSYS HDF5 import interface, a direct evaluation of the actual as-built properties of fiber composite components is made possible. Deviations from the original numerical as-planned design can be imported, and thus, an assessment of the actual performance of the component is enabled. Thus, active monitoring of the real fiber orientation during manufacturing can serve quality assurance purposes. Particularly in the case of manual placement of the reinforcing fiber layers, the effect of a deviation of the fiber angle from the ideal target condition can be evaluated, and the possible scrap can be reduced.

With the system presented here, the individual layers must be recorded manually and individually for each layer and position on the component. This procedure is time-consuming and, in its current form, it is only suitable for special components and high-performance applications. For a transfer of the technology to an industry of broad fiber composite applications, automated measurement and numerical evaluation is essential.

Archiving of the acquired data can not only provide quality assurance according to the as-planned model but can also be used to trace a failure mode back to the manufacturing conditions if the number of components and fracture pattern evaluation is sufficiently high after the component lifetime has been reached. For small businesses and small-scale manufactures, the system enables a system to qualify committee.

Most of the introduced systems, as well as the system presented, rely on expensive hardware and specific software packages and are vulnerable to changes in the environmental conditions. A smaller technological entry level can push a breakthrough of visual quality management systems and provide more industrial manufacturers with the possibility to quantify the condition of their fabricated composite structures.

## Figures and Tables

**Table 1:** Composite properties from the Krempel KGBX 2508 HT 3K prepreg.

Property	Symbol	Value		Std. Deviation		Image
Young’s modulus direction 1 and 2	$E_{11}$	47.58	GPa	1.2	GPa	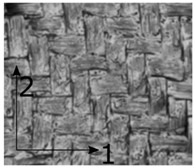
Tensile strength direction 1 and 2	${\sigma_{11}}_{max}$	549	MPa	19	MPa	
Failure strain direction 1 and 2	${\epsilon_{11}}_{max}$	1.14	%	0.03	%	
Shear modulus direction 12	$G_{12}$	4.558	GPa	0.126	GPa	
Shear strength direction 12	${\tau_{12}}_{max}$	106	MPa	3.9	MPa	
Failure strain direction 12	${\epsilon_{12}}_{max}$	5.72	%	1.3	%	

**Table 2:** Layup scheme of the robotic limb.

**Symmetrical Robotic Limb**			
Top shell layup	[45°/−45°, 0°/90°, 90°/0°, −45°/45°]	Lower shell layup	[45°/−45°, 0°/90°, 90°/0°, −45°/45°]
**Unsymmetrical Robotic Limb**			
Top shell layup	[0°/90°]_4_	Lower shell layup	[0°/90°]_4_
Final degree of cure: 0.98		Final fiber volume content: 46%	

## Data Availability

The data presented in this study are available on request from the corresponding author.
